# Challenges to Introducing Integrated Diabetes Care to an Inner-Regional Area in South Western Sydney, Australia

**DOI:** 10.5334/ijic.4692

**Published:** 2020-05-05

**Authors:** Reetu Zarora, Rati Jani, Freya MacMillan, Anna Pham, Ally Dench, David Simmons

**Affiliations:** 1 Macarthur Clinical School, School of Medicine, Western Sydney University, Campbelltown, New South Wales, AU; 2 Nutrition and Dietetics, Faculty of Health, University of Canberra, Australian Capital Territory, AU; 3 School of Health Science, Western Sydney University, Locked Bag 1797, Penrith, New South Wales, AU; 4 Picton Family Medical Centre, Wollondilly, New South Wales, AU; 5 Community and Corporate, Wollondilly Shire Council, Wollondilly, New South Wales, AU

**Keywords:** integrated care, diabetes mellitus, rural health, peer group

## Abstract

**Introduction:**

Diabetes care often requires collaboration between general practitioners, allied health professionals, nurses, and/or medical specialists. This study aimed to describe the establishment of an integrated diabetes prevention and care approach in an area with limited access to primary and secondary care, and the challenges faced in its initial development.

**Description:**

A qualitative research approach to identify challenges was taken. Data included meeting minutes, observational data and reports involving local clinical and non-clinical stakeholders from June 2016- December 2018 and were thematically analysed.

**Discussion:**

Key challenges were low patient attendance in general practice, healthcare professional time, low participation at health promotion activities/peer support groups and diabetes education reflecting a low priority among people with and at risk of diabetes. Coordination between services remained a challenge.

**Conclusion:**

This study highlights the need to integrate new diabetes services with existing health activities in the community and the importance of allowing flexibility and regular contact with local healthcare professional and community to encourage their involvement. Regular meetings with the funders, internal and external stakeholders are key for sustainability and to adapt programmes to the local situation. Further work is needed to identify and implement strategies to overcome these challenges.

## Introduction

Diabetes mellitus is a significant and fast growing health problem in Australia and around the world [1]. Diabetes related complications, such as macrovascular disease, lower limb amputations, blindness and kidney failure, increase the risk of hospitalisation and premature death [2]. In 2017–18, 1.2 million people in Australia were diagnosed with diabetes [3]. Hospital admissions due to diabetes were over 1 million in 2016–2017 (10% of all hospitalisations) [3]. Diabetes related death and hospitalisation rates are two times as high in rural and remote areas as in major cities, independent of socio-economic status [3]. One approach to improving care and reducing hospital costs is for better integration between Australian primary and secondary care [4].

A review of integrated diabetes care across the world [5] concluded that outcomes can be improved with greater integration at the primary, secondary and tertiary level of diabetes care. Such integrated diabetes care requires greater support for self-management through structured education, and diabetes peer support. A recent study in Australia [6] provides further evidence that integrated care is required to close the gap for people with diabetes in the health system. Similarly, a study of the implementation of the chronic care model in a rural primary care practice (in the United States) was associated with improvements in patient outcomes, service provider satisfaction, Haemoglobin A1c, patient knowledge and cholesterol levels when adhering to standards of care [7].

**Figure 1:**
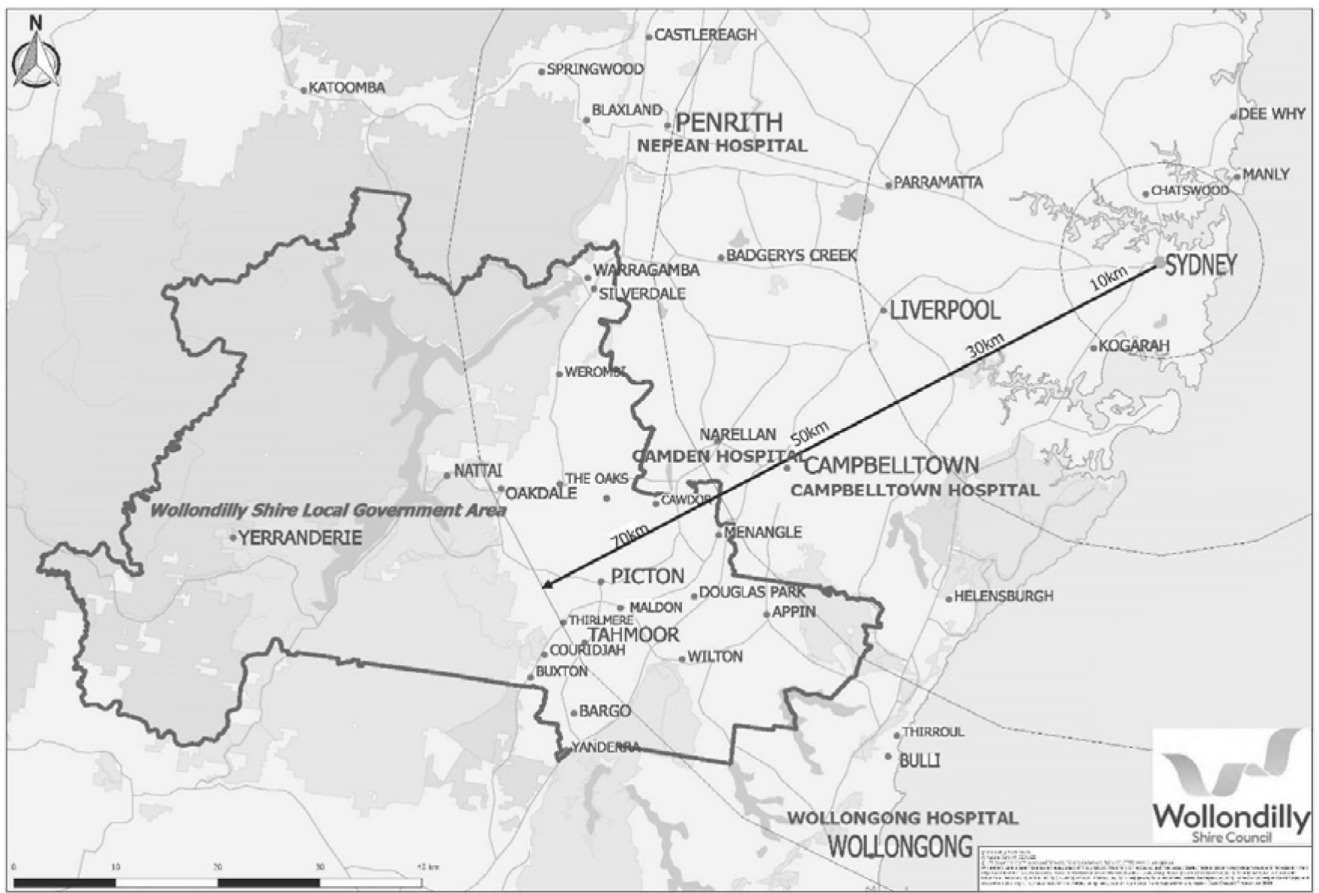
Wollondilly Shrine in New South Wales, Australia and the nearest Local Health District hospitals in adjoining suburbs.

Wollondilly is an inner-regional local government area (2,561 square kilometres) in South Western Sydney, in New South Wales, Australia. The population in 2016 was 48,519, among whom 1,552 were Aboriginal origin dispersed across the district [8,9], and of the total population, approximately 2,519 had diabetes [10]. Wollondilly consists of 17 towns and villages with no secondary care centres, no endocrinologists, one community health centre and few local allied health providers (podiatrists, dietitians, credentialed diabetes educator, pharmacists, and optometrists). There is also limited access to general practice, with a low general practitioner: people ratio of 1:2960. This limited range of health services often requires residents to travel to adjoining local government areas [11]. However, community transport is a challenge in Wollondilly, further limiting access to health services [11]. Figure 1 [12] is a map which shows the Wollondilly Shire in New South Wales, Australia and the nearest Local Health District hospitals in adjoining suburbs.

Being overweight or obese can increase the risk of a person developing type 2 diabetes [13]. Obesity rates in Wollondilly are one of the highest in South Western Sydney [11], with 56% of adult’s overweight or obese and 11% with diabetes or high blood sugar in South Western Sydney [13].

The aim of this paper is to describe the establishment of an integrated diabetes prevention and care approach, the Wollondilly Diabetes Programme (WDP), and to report on barriers faced in its initial development.

## Ethical approval

Ethical approval was granted by Western Sydney University Human Research Ethics Committee (HREC Approval Number- H11826).

## Description of the Wollondilly Diabetes Programme

The Wollondilly Health Alliance was established in 2014 to facilitate joint working between the Local Health District (state funding), the Primary Health Network (Federal funding) and Wollondilly Shire council, to improve health care in the region [14]. The WDP aimed to achieve integrated diabetes care through local functional integration (information technology, clinical services, community systems and strategic planning), and introduction of new and existing online resources for health professionals for optimal patient healthcare and continued professional development. The WDP included (i) clinical services to improve the clinical management of people with type 2 diabetes, aged ≥18 years and occasionally type 1 patients and (ii) health promotion/peer support to support healthy lifestyle and self-management among those with or at risk of type 2 diabetes.

### The Wollondilly Diabetes Programme: organisational framework

The Wollondilly Health Alliance includes a Care process (clinical) and a Health promotion (community) working group. Core membership of the Care process working group includes representatives from general practice, non-government organisations and private industry. Core membership of the health promotion working group includes health promotion officers, project managers/officers and community support workers [14]. The WDP team were invited to the working groups over the first year, and attendance occurred when possible. This meeting was the main process, besides written reports, to ensure the Wollondilly Health Alliance were kept aware of the WDP progress. The WDP team itself met weekly in local council offices to monitor the progress of the programme and discuss the challenges and ways of addressing these. Council staff and the Wollondilly Health Alliance Project Manager (or delegate) also attended at times.

Linkage with other Wollondilly healthcare professionals (besides through example practice based work) occurred through new bi-monthly Clinician Reference Group (CRG) meetings. The focus of these meetings was to share information about the progress of all aspects of the programme (clinical services, health promotion and peer support programme) and to receive feedback and guidance on how the programme should proceed, provide diabetes healthcare professional education (including case based discussions) and to nurture an integrated approach to diabetes care.

### The Wollondilly Diabetes Programme: diabetes clinical services

#### Clinics and group education

**Figure 2:**
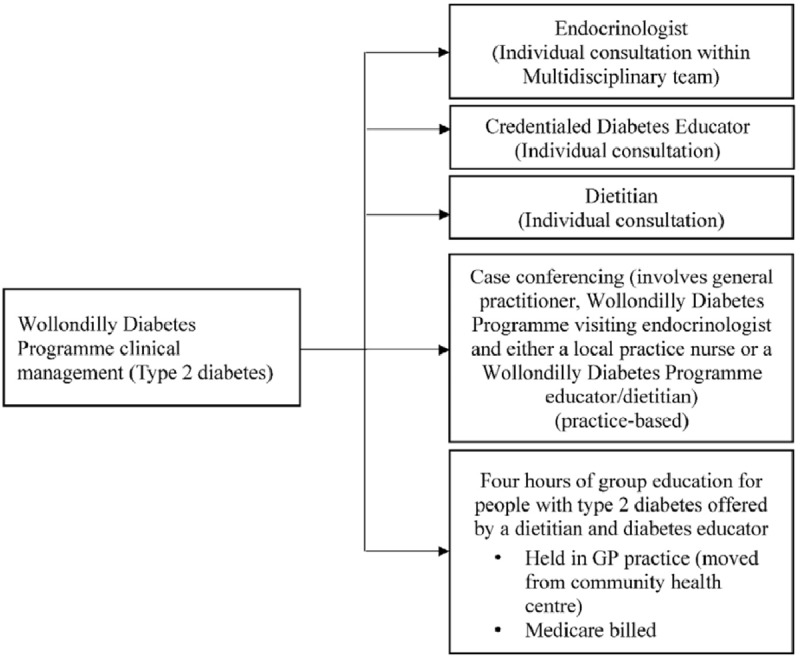
Illustrates the Wollondilly Diabetes Programme clinical management services provided as part of the programme.

The WDP clinical management services are summarised in Figure 2 and included monthly endocrinologist led multidisciplinary clinics, weekly credentialed diabetes educator sessions, weekly dietitian clinics, weekly podiatry screening and weekly group education sessions with an educator and dietitian. Podiatry screening was part of the WDP in year 1. Weekly type 2 diabetes group education sessions were provided by an eligible dietitian and credentialed diabetes educator, either at the Local Health District operated community health centre or within general practices.

#### Case conferencing

Case conferencing allows an endocrinologist to visit general practices to advise general practitioners with managing more complex diabetes patients/cases. The case based discussion is structured for both healthcare professional education and clinical decision-making, and builds a relationship between the specialist service and primary care. [15]. Case conferences were organised with the attendance of the general practitioners, a WDP visiting endocrinologist and either a local practice nurse or a WDP educator/dietitian. The patient was not usually invited (as allowed by Medicare) to maximise the number of cases discussed and to ensure an open and frank discussion about the issues. Cases discussed were those with significant hyperglycaemia (HbA1c ≥9%, 75 mmol/mol), uncertain diagnoses, wider metabolic issues and where next management steps were uncertain. The “patient-free” approach has previously been used in England [16] and it differs from the patient present approach used elsewhere [17].

### The Wollondilly Diabetes Programme: digital health support

HealthPathways is an online referral guidelines portal used by health professionals at the point of care to plan patient care, and where necessary, provide information on how to refer to specialists and other services [18]. The WDP team encouraged general practices to use HealthPathways.

The Australianised Cambridge Diabetes Education Programme (AusCDEP) is a competency based online learning tool for diabetes related topics that supports all levels of healthcare practitioners. For example, it included basic topics (what is diabetes) for non-clinical administrative staff, such as the receptionist, key issues (e.g. hypoglycaemia) for all clinical staff to advanced topics (enteral nutrition and diabetes) for more specialist staff to address the needs and deliver quality care [19]. The WDP piloted this learning tool among general practices in Wollondilly.

### The Wollondilly Diabetes Programme: diabetes prevention

**Figure 3:**
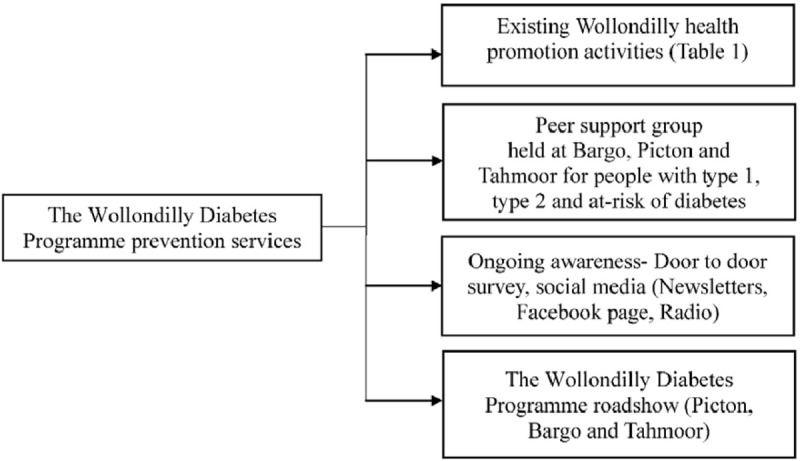
The Wollondilly Diabetes Programme prevention and peer support services.

The aim of this component was to encourage Wollondilly residents to take advantage of new and existing health promotion activities running in Wollondilly. There were several activities promoting healthy eating, lifestyle and social support in Wollondilly initiated by the Wollondilly Health Alliance. The team gained information on all health promotion activities, by attending the bi-monthly health promotion working group meetings. The WDP also initiated diabetes awareness approaches including a roadshow, door to door survey and promotion through social media (Newsletters, Facebook page, Radio). Figure 3 illustrates the WDP prevention and peer support services.

### The Wollondilly Diabetes Programme: peer support

In a peer support programme, one person supports another with similar personal circumstances. Self-management of diabetes can be supported by peer support facilitators who provide guidance and support on ‘how to do’ rather than the ‘what to do’ [20]. Four key peer support components included self-management support, linkage to clinical care, emotional support and ongoing support. Peer support facilitators, who were not health professionals but rather facilitators of discussions, facilitated the peer support groups [21]. Peer support facilitators were invited to attend a two-day training workshop based upon a previously validated peer support facilitator training module successfully followed in a RAandomised controlled trial of Peer Support In Type 2 Diabetes (the RAPSID study) [22]. The WDP team informally and discretely observed peer support facilitators during the workshop. Peer support facilitator skills such as motivational interviewing, active listening, problem solving, and goal setting were practised.

Not all residents attended general practices within Wollondilly and therefore nine recruitment approaches were used to recruit participants, described below in Table 1.

**Table 1:** The Wollondilly Diabetes Programme recruitment approaches.

	Recruitment approaches

General Practices	The general practitioners were encouraged to refer patients to the Wollondilly Diabetes Programme group education sessions and peer support programme.
Wollondilly-wide flyer distribution	The Wollondilly Diabetes Programme flyers were placed at various locations across Wollondilly including medical centres, community centres, food outlets, schools, private business/religious places and leisure centres.
Community engagement at community groups	The Wollondilly Diabetes Programme was promoted at regular and one-off events organised by the Wollondilly council, New South Wales Health. Examples of community groups included: Men’s shed, community vegetable gardens, community pantry. Existing Wollondilly Diabetes Programme participants were offered the opportunity to be part of the peer support programme, when they were consulted by either Dietitian or Credentialed Diabetes Educator for individual consults and when attended group education
Social Media- Newspapers/Radio	The Wollondilly Diabetes Programme team approached local newspapers, radio stations, magazines and Public School Newsletters, to advertise the Wollondilly Diabetes Programme.
Social Media-online	A Wollondilly Diabetes Programme Facebook page was created and Wollondilly-associated Facebook community and council pages were sent a request, to post the Wollondilly Diabetes Programme
Word of mouth	The entire Wollondilly Diabetes Programme team encouraged existing interested participants to let their network of friends, family, colleagues know about the Wollondilly Diabetes Programme, and encourage their network to contact the Wollondilly Diabetes Programme over the phone, in-person or via email for participation or more information.
Door to door survey	Door knocking is being undertaken across Wollondilly and commenced in February 2017. Flyers are distributed while conducting surveys. So far 5418 flyers have been distributed.
The Wollondilly Diabetes Programme road show	The aim of this strategy is to invite patients for screening at one centralised location. The Wollondilly Diabetes Programme road show consisted of the Wollondilly Diabetes Programme team- the endocrinologist, dietitian and credentialed diabetes educator, non-clinical staff and the Wollondilly Diabetes Programme Peer Support Facilitators (already trained) to encourage the attendees to join as peer support facilitators or as peers.
Promotion via peer support facilitators	Residents (peers) with and at-risk of diabetes are encouraged by their fellow community members (peer support facilitators), to join the programme

### Methods: data collection

This qualitative study was conducted in Wollondilly Shire to identify the challenges faced during the programme. It is a prospective observational study using an ethnographic methodology. Data were captured and collated from (Table 2) – Clinician Reference Group meeting notes (n = 2), meeting minutes from weekly WDP meetings (n = 93), individual reports (n = 6) from health professionals and written notes from the participant observer (RZ), a Doctor of Philosophy student with experience in data collection and analysis. One author (RZ) observed and interacted with participants at meetings and documented key words by hand and transcribed soon after to maintain accuracy and for detailed description to explore and examine participants, processes and cultures [23]. Discussions were not voice recorded at meetings but minutes were used instead.

**Table 2:** Illustrates the data sources.

Source	Data *(n)*

Wollondilly Diabetes Programme (weekly) meeting	93 meeting minutes
Clinician Reference Group meeting (held bi-monthly)	2 meeting notes (17 meetings) *
Individual reports from Wollondilly Diabetes Programme clinical and non-clinical staff.	6
Ethnographic approach	Observation at weekly meetings and activities/events when organised and held.

* Notes were taken in only two Clinician Reference Group meetings.

#### Participants

Study participants included both internal (endocrinologists, dietitian, credentialed diabetes educator and health promotion staff) and external stakeholders (general practitioners, practice nurses, pharmacists and allied health and health promotion professionals) who attended meetings.

#### Analysis

Data were thematically analysed by identifying patterns (themes) [24], managed using QSR NVivo 11 Pro software and following Braun and Clarke’s six phase process to thematically analyse qualitative data [24].

##### Familiarisation (Phase 1)

This first phase included becoming accustomed with data. Data were integrated and were closely read multiple times before generating codes by one author (RZ). Data were discussed with co-author (FM) by sharing each other’s perceptions to gain better understanding.

##### Generating codes (Phase 2)

Initial codes were generated by systematically reading and rereading the data to become more engaged with the data. Codes in the narrative of data were labelled on minutes/notes, resulting in a variety of codes. Coding was cross-checked by a researcher (FM) with extensive experience in qualitative research, to assure accuracy and consistency.

##### Searching for themes (Phase 3)

Similar codes were organised together and potential themes were explored and built. Extracts related to the codes were reread to explore if they fit better elsewhere or into the existing theme to prevent any contradictions and continue distinction between themes, expressing a story about each aspect of the data clustered. All relevant data were explored to represent data precisely.

##### Revising and Defining themes (Phase 4 and 5)

This phase included final reviewing of themes to identify any linkage. Each theme with its coded and clustered data were reviewed to avoid conceptual overlap. Themes were agreed upon and were identified which reflected participant’s perspectives on the challenges to implementing the WDP.

##### Report production (Phase 6)

This final phase involved revising the theme names, coding, and all over dataset before final analysis and writing up findings. Final finding are explained in the results section of this paper.

A 15-point checklist was used to ensure trustworthiness of data and to confirm that all six phases were followed (Supplementary table S1) [24].

### Qualitative Results

Participants of the observational study included endocrinologists (n = 2), dietitians (n = 2), a credentialed diabetes educator (n = 1), health promotion professionals (n = 5), general practitioners (n = 8), practice nurses (n = 4), pharmacists (n = 5), and other allied health professionals (n = 4) (a total 31 participants who attended various meetings). Three major themes with were identified from the data collected.

#### Theme 1- Challenges with general practices

All the general practices (total 11 practices) in Wollondilly were approached to work with the WDP clinical services. Two practices considered themselves self-sufficient about diabetes care and declined invitations to participate in clinical care but attended meetings. In the larger practices in Wollondilly, practice nurses were responsible for identifying suitable patients for consultations. Occasionally they were unable to prepare patients for the next month due to their time commitments. This preparation work was often completed outside of their scheduled working hours, which affected the progress of the programme and the patients who were in need of consultation.

Case conferencing was sometimes challenged by the need for a third healthcare professional to attend and variable attendance by practices when patient load was high. Some practices were open only 1–2 days per week and therefore had little time for case conferencing. Availability of the endocrinologist was also an issue, with less than one funded day per week. The duration of case conferencing was also an issue as all session times must match and were to be negotiated at the time of the phone call to the practices.

#### Theme 2- Challenges with allied health

The dietitian and the credentialed diabetes educator worked part-time (two days a week) and not throughout the week, which affected the patient appointment bookings due to their limited availability. Most of the patients preferred consultation or group sessions on weekends or late evenings over weekdays, as they travel to work outside of Wollondilly during weekdays. Even after the services were delivered within the practice, with invitations sent from the practice, patient attendance was low which could be due to low patient priority for diabetes education. Smaller practices were unable to physically accommodate group education sessions. The Medicare funded Chronic Disease Management item [25] limits resulted in some allied health/practices seeing the WDP as a competitor, either by using up one of the 5 available Medicare item numbers, or replacing an income generating appointment. A podiatrist was funded in the first year of the WDP to increase access to podiatry. However, early discussions indicated that this could place the existing private podiatry at financial risk, so only podiatry screening programme was established. After discussions with practice nurses and even general practitioners, one of the barriers to foot screening identified was the stigma associated with feet. Patients were interested more in foot treatment (toe nail cutting), as opposed to foot screening alone.

#### Theme 3- Challenges with community participation

The peer support programme and community engagement activities/events were slower to set up than clinical services. It was a challenge to engage with residents of all the towns due to their geographical dispersal (distance between towns and distance between individual houses). Most of the events and peer support activities were initially held in two major towns of Wollondilly and only a few residents of other towns and villages would attend due to diabetes education being a low priority and the distance and travel time, with residents having to travel for at least 15–35 minutes and for some for up to an hour. It was challenging to conduct surveys and to interact with residents in certain towns of Wollondilly as the properties are large and gated, which made communication with these residents difficult. The WDP was promoted through existing community networks within Wollondilly but their available time and detailed knowledge for promotion were limited.

We experienced similar challenges to reach the population living in semi-rural or rural areas as noticed in other studies, such as low attendance and low priority for diabetes education [26,27,28]. The issue of transport disadvantage is identified for semi-rural and rural areas in Australia [29], which relates to the challenge of accessing healthcare noted in our study. It specifically affects people from lower socioeconomic background and older people who are unable to drive.

Data collected such as the WDP services uptake, awareness of the WDP through door-to-door survey, number of people joining peer support group was used as a quality improvement tool and a clinical audit of the programme is ongoing to find the impact of the programme on population. The numbers were reviewed every week during the team meetings (Table 3).

**Table 3:** Attendance & uptake data.

Attendance data			

Variable	Key questions	Measure	Resource

Wollondilly Diabetes Programme-Organisational support	Number of health promotion working group meetings attended by the Wollondilly Diabetes Programme team. Number of clinicians attending vs invited to clinician reference group meeting. Number of general practices completing vs invited for AusCDEP modules for continued professional development.	Health promotion working group meetings- 7/7. Number of clinicians invited vs attending- 12/38. Number of general practices invited vs completing AusCDEP- 1/11	By 1–2 staff routinely (Health promotion and administration staff)
Wollondilly Diabetes Programme-Clinical support	Number of general practices participating in the case-conference. Number of patients (>18 years) attending multidisciplinary clinic (appointments attended) vs number of appointments booked for each service until December 2018.	General practices participating -3/11 Diabetes Educator- 123/157 Endocrinologist- 56/88 Diabetes group education-113/197 Dietitian- 162/214 Podiatry screening (until June 2017)- 41/47	By 1–2 staff routinely (Health promotion and administration staff)
Wollondilly Diabetes Programme-Peer support	Number of participants completed the peer support facilitators training workshop. Number of peers participating in the Wollondilly.	Participants completed the peer support facilitators training workshop – 5 Peers- 25	By 1–2 staff routinely(Health promotion and administration staff)
Wollondilly Diabetes Programme-Health Promotion	Number of Wollondilly Diabetes Programme interactions at promotion at various health promotion community activities ongoing/one-off in Wollondilly.	Number of interactions is 1280 (from November 2016-December 2018)	By 1–2 staff/students routinely(Health promotion and administration staff)
Door to door survey and data collection	People agreed to complete the diabetes record questionnaire The number of door to door surveys completed in total.	Diabetes record questionnaire-37/250 (14.8%). 619/4418 (14%) for 1968 residents have been surveyed until October 2018.	By 1–2 staff/students routinely (Health promotion and administration staff)

## Discussion- Lessons learned from the case

This study describes in detail the key challenges and lessons learned while implementing an integrated diabetes care and prevention service de novo into an inner-regional area. Most of the following lessons learned may help future projects to increase their integrated care programme/project efficacy and effectiveness.

### Engage regularly with the local healthcare professionals from the beginning to reach the target population, allow flexibility encouraging them to participate

Creating the relationship with primary care was of key importance with the need to overcome the initial hump of work to generate much-needed referrals to specialist endocrinologist, allied health clinics and for case conferencing. Some practices open only 1–2 days per week and therefore had little time for case conferencing. We approached all practices and encouraged them to participate. Seven practices (7/11) participated in at least one aspect of the programme. The limit of 5 Chronic Disease Management Medicare items resulted in some allied health/practices seeing the WDP as a competitor. Therefore, podiatry treatment never started and screening was no longer provided to avoid any conflicts. General practices expressed that the systematic referral processes were time consuming to implement, as the process required identifying patients systematically. Time constraints resulted in fewer referrals than expected in spite of the clinical burden (that is clinical inertia) It has been known for over 20 years that diabetes care and patient satisfaction can be improved, by providing support to primary healthcare teams [30] and the WDP is another example of this approach.

Joint specialist and general practitioner case conference evaluations in Western Sydney and Hunter and New England have been associated with improvements in clinical parameters such as Haemoglobin A1c, Body Mass Index and blood pressure of patients. They have high program acceptability from both general practitioners and patients [17,31]. Such Australian integrated diabetes care approaches, along with the Beacon model (diabetes care within general practice supported by an endocrinologist, credentialed diabetes educator and delivered by advanced skill general practitioners) show that better glycaemic control is achievable through a more integrated approach between general practitioners and specialist care [32].

### Effective change management to tackle challenges, improve productivity and collaboration, without deviating from the integrated care principles

Change management made implementation sustainable when services were delivered in practices avoiding competition. The initial plan was to provide podiatry services, however this was terminated to avoid conflicts with the local allied health. Group education sessions were moved to practices to build rapport with the practice staff and overcome such perception. The WDP dietitian and credentialed diabetes educator contacted patients with type 2 diabetes at the Picton and Wilton practices for the group education. This was useful, and increased numbers of attendance, as often due to staffing issues the individual practice did not have time to call patients. The idea to move the education to individual practices was also to increase trust with patients as it was within their GP practice and to combat the lack of transport for some. Interventions at health service provider level also has shown to have a positive impact on the overall health and lifestyle of patients with type 2 diabetes living in a rural area (Alberta, Canada) including an increase in satisfaction level of both patient and the provider [33].

There were changes to the WDP team in the second year (dietitian and administration staff); however, new roles improved the productivity of certain aspects of the programme (such as community coach for peer support programme), volunteer involvement improved the health promotion aspect of the programme, increasing the community participant and awareness on the WDP. Leadership plays a significant role for successful implementation to address population needs, setting clear goals for the team, managing changes and adding innovative designs to an integrated programme [34,35]. The changes were successfully managed under the strong operational leadership; responsibilities were distributed within the team to manage different aspects of the programme, who were provided continuous support and guidance.

### Community participation is key in health promotion activities; regular contact can help deliver messages through various activities/events

A needs assessment consultation process conducted by the Wollondilly Health Alliance reported the community, general practitioner, allied health provider and non-government organisation perceptions of the health services in Wollondilly. Challenges in accessing health services were identified by nearly 60% of survey respondents because of the shortage of general practitioners, resulting in long wait times to see a general practitioner across Wollondilly [11].

Challenges with community participation included slower set up of prevention activities and low attendance sometimes reportedly due to distance and travel time but also a lack of prioritising diabetes education by patients. Community activities/events were initially mostly held in the two largest towns (Picton and Tahmoor) of Wollondilly and residents from other towns had to travel long distances (15–35 minutes) to these towns and health service centres, worsened by poor public transport in Wollondilly. Limited public transport was also noted as one of the challenges to a diabetes prevention programme (Greater Green Triangle Diabetes Prevention Project) in a rural area of south eastern Australia [36]. Promotion of the WDP was also through existing networks within Wollondilly but their available time and detailed knowledge for promotion were limited. This was addressed by increasing the presence of the WDP through students and staff (time consuming) attendance at community events to promote the programme. We collaborated with the Dilly Wanderer to promote the WDP, an outreach van travelling to different towns and villages in Wollondilly [37].

### Regular contact with the external stakeholders/funders could inform them about what else may be needed to achieve the aims/results

It was not possible to have a joint meeting with all working groups at one time mainly due to time restrictions and other commitments. Separate meetings were held with community workers and council. The need for joint working between organisations, inter-professional collaboration, patient awareness and adequate and long-term financial support required a major shift in the ‘natural’ modus operandus. Staffing issues limited the practice opening times and the inclusion of a third health care professional for case conferencing.

Case conferencing was initially a part of the WDP however; after one year, the contract for this service shifted from the Wollondilly Health Alliance to the district-wide contract with the local Primary Health Network. A different specialist was extended to work in Wollondilly who was linked with the hospital clinic in Campbelltown (adjoining suburb/area), but not linked to the clinical aspects of the WDP. As a result, the WDP allied health professionals could no longer readily link to case conferencing. This led to a reduction in the number of patients seen in the WDP clinics. The change in funding came from decisions by the Local Health District; at a district wide committee level not through the Wollondilly Health Alliance committees. The funding arrangement and responsibilities within the system are complex, as they are spilt between all levels of government. In Australia, most hospital care is provided through state government systems, but ambulatory care (both primary and secondary) is funded by the federal government through Medicare (a partly to fully publicly funded fee for service system) [38]. As a result, functional integration is problematic, with parallel systems in place between State, Federal and Local governments.

### Regular meetings are significant in mapping the progress and to timely address the challenges

We could identify and address some of the challenges from the beginning of the programme because of the regular team meetings. Shared clinical priorities improved integrated care delivery through working group meetings and incentives encouraged participation of general practitioners such as for case conferencing. Continuous professional development platform was offered through Clinicians reference group meetings to support joint working, and innovation for better care delivery. Data were used as a quality improvement tool to help identify and address any gaps. Regular meetings with both internal and external stakeholders were key in addressing the challenges and increased community participation.

### Strengths of this study

We collected data based on human experience and included the feedback/comments from clinical and non-clinical staff who participated in the programme. The challenges faced have been examined throughout the duration of the delivery of the programme, so that adjustments/solutions can be identified promptly to address these. Meeting minutes were collected from the start of the programme, which allowed us to understand the barriers from programme initiation. A second researcher independently verified qualitative data coding to ensure the trustworthiness of this analysis. A major strength of the study is the transferability of the lessons regarding the challenges, and the key interventions (e.g. case conferencing and clinician reference groups which are being taken district-wide, general practice based group education and peer support which have been extended to other areas and the community multidisciplinary clinic which improved access to a diabetes specialist team).

### Limitations of this study

A limitation of this study is that it only includes the views of health professionals involved in the programme and lacks patient feedback regarding their view on diabetes care delivered in their area. Interviews with patients and other stakeholders are planned, which should inform further enhancements to the programme. There was inconsistency in attendance at meetings and some attendees might not have provided in-depth information on highlighted issues, affecting the interpretation of the collected data. The study only covered an inner regional area and hence, does not have national generalisability. A further limitation is that only the processes and challenges have been presented. Outcome studies are underway, separately evaluating the clinical, case conferencing and health promotion components.

## Conclusion

This study has described the implementation of the WDP along with the challenges faced at various stages and found that to provide seamless integrated diabetes care, both external and internal stakeholders need to work closely, but coordination and joint working requires additional time and supportive systems. Significant challenges occurred, in spite of substantial need, and the alignment of the WDP with the goals of ‘The Australian National Diabetes Strategy 2016–2020’ including improving the co-ordination of resources by all levels of government [39]. Major hurdles are financial barriers, time and human resource constraints, and inter-organisational issues at every level. The National Association of Diabetes Centres has recently developed a practical toolkit that lists a range of implemented diabetes models of care in clinical settings across Australia for the management of type 2 diabetes [40]. The WDP incorporated several of the approaches adopted, but also differed in providing support at multiple levels from patient, healthcare team to community, which was an innovative approach.

While the WDP aimed to provide better access to diabetes care by inter-professional and inter-organisational collaboration, a fuller, quantitative evaluation is required to assess the extent to which care was improved, and its associated costs. Further work is also needed to gather in depth perspectives on the challenges identified, to help find strategies to overcome these barriers. This implementation approach can be applied to similar geographical regions and population spread in other countries. Considering the lessons learned from this integrated care case could guide the development of an efficient approach in a similar setting.

## Additional File

The additional file for this article can be found as follows:

10.5334/ijic.4692.s1 Supplementary table S1. A 15-point checklist of criteria for good thematic analysis.
