# Supplementary material for: Challenges to Introducing Integrated Diabetes Care to an Inner-Regional Area in South Western Sydney, Australia
Source: Int J Integr Care. 2020 May 5;20(2):6. doi: 10.5334/ijic.4692 (PMC7207248; doi:10.5334/ijic.4692)
Supplement: Supplementary table S1. — A 15-point checklist of criteria for good thematic analysis. [file ijic-20-2-4692-s1.pdf]

Supplementary table S1

Process	No.	Criteria	Reported in manuscript
Transcription	1	The data have been transcribed to an appropriate level of detail, and the transcripts have been checked against the tapes for 'accuracy'.	**No- there were no tapes/discussion recorded**
Coding	2	Each data item has been given equal attention in the coding process.	**Yes**
	3	Themes have not been generated from a few vivid examples (an anecdotal approach), but instead the coding process has been thorough, inclusive and comprehensive.	**Yes**
	4	All relevant extracts for all each theme have been collated.	**Yes**
	5	Themes have been checked against each other and back to the original data set.	**Yes**
	6	Themes are internally coherent, consistent, and distinctive.	**Yes**
Analysis	7	Data have been analysed - interpreted, made sense of - rather than just paraphrased or described.	**Yes**
	8	Analysis and data match each other – the extracts illustrate the analytic claims.	**Yes**
	9	Analysis tells a convincing and well-organised story about the data and topic.	**Yes**
	10	A good balance between analytic narrative and illustrative extracts is provided.	**Yes**
Overall	11	Enough time has been allocated to complete all phases of the analysis adequately, without rushing a phase or giving it a once-over-lightly.	**Yes**
Written report	12	The assumptions about, and specific approach to, thematic analysis are clearly explicated.	**Yes**
	13	There is a good fit between what you claim you do, and what you show you have done – i.e., described method and reported analysis are consistent.	**Yes**
	14	The language and concepts used in the report are consistent with the epistemological position of the analysis.	**Yes**
	15	The researcher is positioned as *active* in the research process; themes do not just 'emerge'.	**Yes**
